# Decoding the HIF-1-driven metabolic-inflammatory-immune axis in sepsis-associated lung injury: a comprehensive overview

**DOI:** 10.3389/fimmu.2025.1658103

**Published:** 2026-01-05

**Authors:** Shuang Zhang, Beilin Hu, Yuang Fang, Mudi Liu, Qingmei Liu, Ye Chen, Jun Zhou

**Affiliations:** 1 Department of Anesthesiology, Geriatric Diseases Institute of Chengdu/Cancer Prevention and Treatment Institute of Chengdu, Chengdu Fifth People’s Hospital (The Second Clinical Medical College, Affiliated Fifth People’s Hospital of Chengdu University of Traditional Chinese Medicine), Chengdu, Sichuan, China; 2 Department of Anesthesiology, The Affiliated Hospital, Southwest Medical University, Luzhou, Sichuan, China; 3 Anesthesiology and Critical Care Medicine Key Laboratory of Luzhou, Southwest Medical University, Luzhou, Sichuan, China; 4 Department of Traditional Chinese Medicine, The Affiliated Hospital, Southwest Medical University, Luzhou, Sichuan, China

**Keywords:** HIF-1, immunity, lung injury, metabolic reprogramming, sepsis

## Abstract

Sepsis is a systemic inflammatory response syndrome triggered by infection that frequently involves multiple organs, ultimately leading to multiple organ failure. Among affected organs, the lungs represent the most vulnerable target. Sepsis-associated lung injury (S-ALI) is a common critical illness that can progress to acute respiratory distress syndrome in severe cases, resulting in high morbidity and mortality. Currently, clinical management relies predominantly on mechanical ventilation and supportive care, as no specific pharmacological treatment exists for S-ALI. The pathogenesis of S-ALI is characterized by uncontrolled inflammation, microcirculatory dysfunction, immune dysregulation, mitochondrial impairment, and oxidative stress. Notably, mitochondrial dysfunction and oxidative stress are closely associated with tissue hypoxia and metabolic reprogramming. Hypoxia-inducible factor-1 (HIF-1) is a pivotal transcription factor that regulates gene expression under hypoxic conditions. It becomes activated during hypoxia and inflammatory responses, thereby coordinating cellular metabolic adaptation and inflammatory pathways. In S-ALI, both the expression and activity of HIF-1 are markedly upregulated, playing a critical role in modulating inflammation, immunity, and metabolic reprogramming. These findings suggest that targeted modulation of HIF-1-mediated metabolic reprogramming in S-ALI may improve patient outcomes by simultaneously addressing inflammatory, immune, and metabolic dysfunction. This review examines the pathogenesis of S-ALI, HIF-1-mediated metabolic reprogramming in S-ALI, the crosstalk between HIF-1 and multiple signaling pathways, and its impact on inflammatory responses and immune function. Our goal is to identify novel therapeutic targets for S-ALI treatment.

## 1 Introduction

Sepsis is an acute, life-threatening condition characterized by a dysregulated host response to infection, typically caused by bacteria, viruses, or other pathogens. This dysregulation leads to potentially fatal organ dysfunction. The pathogenesis of sepsis involves an excessive immune response that triggers systemic inflammatory response syndrome (SIRS), subsequently progressing to multiple organ dysfunction syndrome. This cascade makes sepsis a significant global health burden (1). Each year, sepsis claims millions of lives worldwide. A landmark 2017 study revealed that sepsis accounts for 19.8% of global deaths, affecting approximately 11 million people (95% confidence interval: 10.1–12 million). Moreover, its incidence continues to rise in parallel with increasing infection rates (2) and population aging.

The onset of sepsis is frequently preceded by infections, particularly bacterial infections. The infection-triggered inflammatory response initiates a cascade of events, including cytokine release, increased vascular permeability, and hemodynamic alterations. These changes ultimately result in organ ischemia and dysfunction (3). Epidemiological data indicate that approximately 50% of septic patients develop acute lung injury, which may progress to acute respiratory distress syndrome. Furthermore, acute lung injury represents one of the leading causes of mortality in septic patients (4). Patients with sepsis-associated lung injury (S-ALI) typically present with hypoxemia resulting from impaired gas exchange and pulmonary edema secondary to inflammatory exudation. At the molecular level, S-ALI pathogenesis is highly complex, encompassing inflammatory dysregulation, programmed cell death, barrier disruption, immune dysfunction, and mitochondrial metabolic disturbances (5, 6).

During the inflammatory response, cellular energy metabolism intensifies, leading to increased oxygen consumption and reactive oxygen species production. This creates an imbalance between oxygen supply and demand, resulting in relative tissue hypoxia and progressive mitochondrial dysfunction. As inflammation intensifies and accumulates within the lungs, pulmonary epithelial cells are damaged and the endothelial barrier is compromised, allowing significant fluid extravasation that causes pulmonary edema. Lung tissue injury triggers neutrophil migration and alveolar macrophage activation. These activated immune cells release additional cytokines that amplify the inflammatory response and further damage type II alveolar epithelial cells. Consequently, surfactant is inactivated, lung compliance decreases, and atelectasis develops, collectively exacerbating respiratory dysfunction. Persistent pulmonary inflammation disrupts the ventilation-perfusion ratio and intensifies refractory hypoxemia. Unfortunately, the diagnosis of S-ALI is often delayed due to the absence of definitive diagnostic criteria. Early identification and intervention are critical for improving patient outcomes. Currently, effective treatments remain limited, with management primarily focused on symptomatic relief.

In septic states, the body experiences relative hypoxia accompanied by mitochondrial dysfunction, primarily manifested as impaired oxidative phosphorylation and enhanced anaerobic glycolysis. The development of S-ALI further aggravates systemic hypoxia. Hypoxia-inducible factor-1 (HIF-1) serves as a crucial transcription factor and regulatory protein that enables cellular adaptation to hypoxic conditions. First discovered in 1992, HIF-1 is an oxygen-dependent transcriptional activator (7). It is ubiquitously expressed across tissues and plays a pivotal role in maintaining oxygen homeostasis by regulating the expression of numerous hypoxia-responsive genes (7). Recent reports suggest that HIF-1 plays a significant role in various pulmonary diseases, including inflammation, immune regulation, and fibrosis promotion (8–10), through cytokine modulation. However, the underlying molecular mechanisms remain incompletely understood. This review aims to examine the role of HIF-1 in S-ALI pathogenesis and to explore HIF-1-targeted preventive and therapeutic strategies for S-ALI, thereby providing a foundation for improved clinical management of septic patients.

Methods: We conducted a comprehensive literature search in the PubMed database using the keywords “HIF-1,” “sepsis,” “lung injury,” “metabolic reprogramming,” “immunity,” and “inflammation.” All relevant articles and reviews published since database inception were retrieved and screened to identify studies addressing the role of HIF-1 in the pathophysiological processes of S-ALI.

## 2 Pathophysiology of sepsis-induced lung injury

Clinically, patients with sepsis may experience three distinct inflammatory and immune states. In the early inflammatory phase, pro-inflammatory mediators are dramatically elevated, placing the body in a state of cytokine storm. During this stage, large numbers of neutrophils are recruited, and macrophages become activated and polarize toward the M1 phenotype, further propagating inflammation and exacerbating tissue and cellular damage. Concurrently, the immune system mounts a robust immune response. T cells and macrophages phagocytose substantial amounts of necrotic cells while simultaneously undergoing apoptosis as they eliminate inflammatory mediators. Subsequently, the body transitions into an immunosuppressive state through humoral immune regulation, promoting neutrophil reverse migration and macrophage polarization toward the M2 phenotype, thereby counteracting the inflammatory response (11).

**Figure 1:**
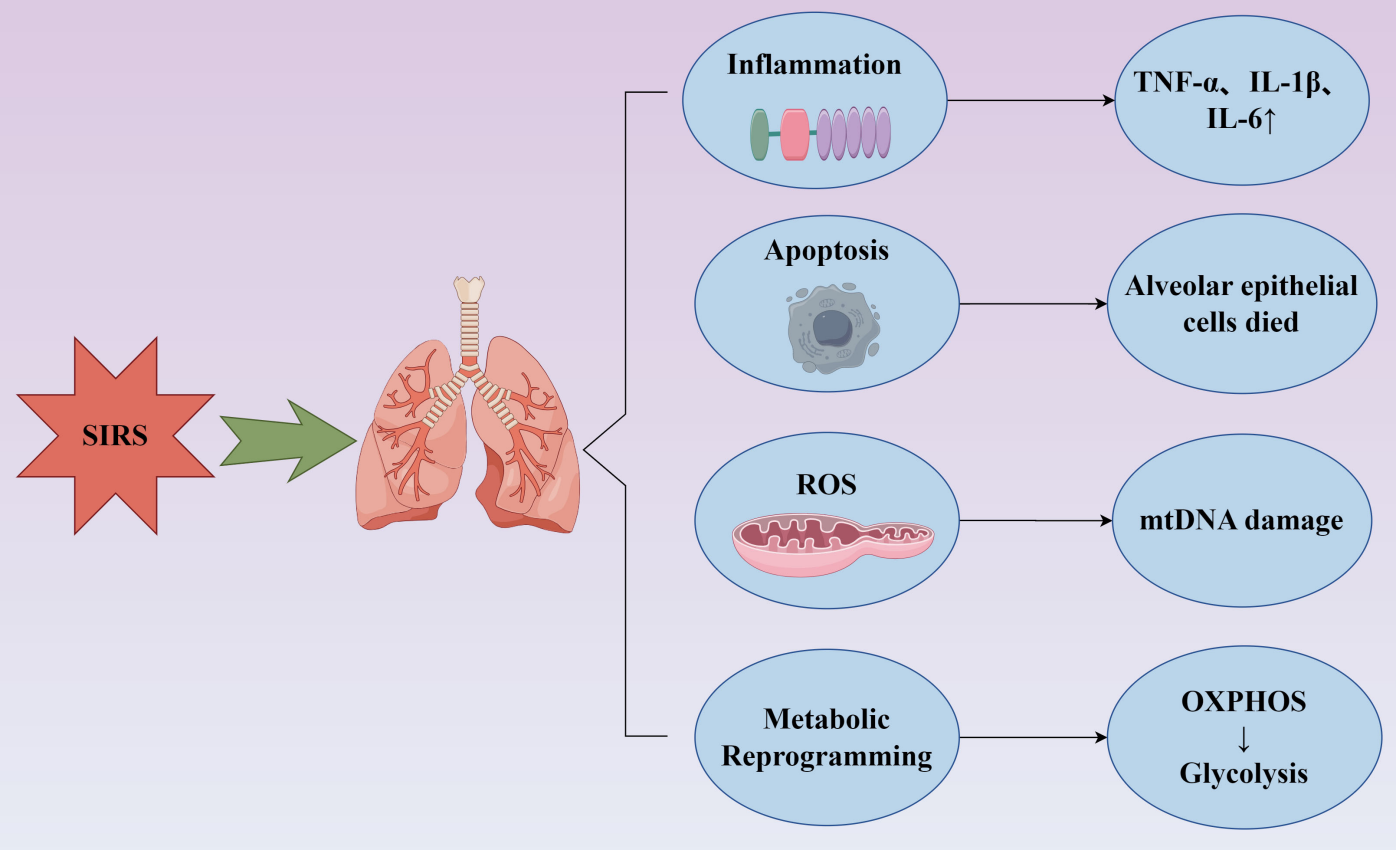
Pathophysiological processes of S-ALI. Infection induces SIRS, which further leads to pulmonary injury through the following mechanisms: exacerbation of inflammation with increased release of inflammatory cytokines such as TNF-α, IL-6, and IL-1β; cellular apoptosis, exemplified by extensive alveolar epithelial cell death; oxidative stress response characterized by ROS release and mtDNA damage; and metabolic reprogramming of tissue cells, featuring impaired oxidative phosphorylation and a shift toward anaerobic glycolysis.TNF-α, tumor necrosis factor-α; IL-1β, interleukin-1β; IL-6, interleukin-6; ROS, reactive oxygen species; mtDNA, mitochondrial DNA; OXPHOS, oxidative phosphorylation.

When pro-inflammatory and anti-inflammatory mediators become imbalanced with pro-inflammatory factors predominating, systemic inflammatory response syndrome (SIRS) develops. Conversely, when anti-inflammatory mediators are excessively released, leading to immunosuppression (12),compensatory anti-inflammatory response syndrome (CARS) emerges (13). When SIRS and CARS coexist and interact to produce clinical manifestations, this is characterized as mixed antagonist response syndrome (MARS) (12–15). Clinically, these three states represent different phases in the sepsis continuum. S-ALI occurring during SIRS at the peak of inflammation directly impacts the prognosis of septic patients. Its pathophysiological basis primarily encompasses inflammatory responses, cellular injury and death, oxidative stress, metabolic reprogramming, and related processes (**Figure 1**).

## 3 Regulation of S-ALI by HIF-1

### 3.1 HIF-1 structure and function

HIF-1 is a heterodimer composed of HIF-1α and HIF-1β subunits, with HIF-1α serving as the functional subunit that regulates HIF-1 activity. The expression and activity of HIF-1α determine the overall function of HIF-1 (16). The HIF-1α subunit is highly sensitive to changes in oxygen concentration; therefore, HIF-1 function and activity are regulated by tissue oxygen levels (16). Under normoxic conditions, the oxygen-dependent degradation domain (ODDD) of HIF-1α undergoes hydroxylation by prolyl hydroxylase (PHD), subsequently binding to the von Hippel-Lindau (pVHL) ubiquitin E3 ligase. This leads to HIF-1α degradation through the ubiquitin-proteasome system, while factor-inhibiting HIF (FIH) simultaneously hydroxylates HIF-1α, inhibiting its transcriptional activity.

However, under hypoxic conditions, the activities of PHD and FIH are markedly reduced, thereby inhibiting the HIF-1α degradation pathway. Consequently, HIF-1α accumulates and translocates to the nucleus, where it dimerizes with HIF-1β (17). The carboxy-terminal transcriptional activation domain of HIF-1α specifically binds to hypoxia response elements (HREs) in target gene promoters, subsequently activating transcription of multiple downstream genes, including vascular endothelial growth factor (VEGF), erythropoietin (EPO), and inducible nitric oxide synthase (iNOS) (18). This cascade triggers a series of adaptive responses that enable tissues and cells to tolerate hypoxic conditions.

### 3.2 HIF-1 and inflammatory response in S-ALI

In the early stages of sepsis, excessive inflammatory responses represent a major cause of acute lung injury (19). Following lung injury, inflammatory pathways such as TLRs/NF-κB (20) and NLRP3/Caspase-1 (17) become activated, recruiting neutrophils to migrate to the injury site and activating macrophages to polarize toward the M1 phenotype. These M1 macrophages secrete abundant pro-inflammatory cytokines, including TNF-α, IL-6, and IL-1β. Studies have demonstrated that these inflammatory cytokines can activate HIF-1α transcription and significantly promote HIF-1 expression (21). Moreover, these mediators increase pulmonary microvascular permeability, compromise endothelial barrier integrity, and cause substantial fluid extravasation into lung tissue, resulting in pulmonary edema. This impairs gas exchange function and further exacerbates tissue hypoxia. Hypoxia itself prevents HIF-1α degradation, thereby promoting HIF-1 synthesis and expression.

Research has shown that TNF-α can activate HIF-1α under inflammatory conditions, while hypoxia stabilizes HIF-1α expression and promotes HIF-1 synthesis, thereby activating target genes and altering alveolar-capillary barrier permeability. Additionally, HIF-1α can cooperate with NF-κB (22), binding to regulatory regions of inflammatory mediator genes to recruit neutrophils and other inflammatory cells, which release inflammatory mediators that amplify the inflammatory response (23).

### 3.3 HIF-1 involvement in cellular injury and death in S-ALI

During the acute phase, the alveolar epithelium sustains extensive damage, and alterations in intercellular junctions impair pulmonary edema clearance. Cytokines released by recruited neutrophils and activated macrophages further aggravate damage to, and even cause death of, alveolar type II epithelial cells. This results in surfactant inactivation, reduced lung compliance, and atelectasis, collectively leading to respiratory dysfunction. Furthermore, S-ALI induces tissue remodeling in later disease stages. As inflammation resolves, pulmonary fibrosis and other sequelae may develop, compromising long-term respiratory function.

HIF-1 can enhance glucose metabolism in airway epithelial cells, thereby improving lung ventilation and ameliorating pulmonary edema and respiratory distress. However, accumulating evidence indicates that HIF-1 promotes apoptosis of alveolar type II epithelial cells (24). Hypoxia inhibits alveolar epithelial cell proliferation and promotes AT-II cell apoptosis by activating the HIF-1α/HRE axis, with the mechanism involving Bnip3L (25). Therefore, targeting HIF-1α may represent a novel strategy to attenuate acute lung injury. Notably, HIF-1 activation exhibits cell type-specific effects during S-ALI progression: in early stages, it regulates inflammation, vascular leakage, and remodeling in immune and endothelial cells; during progressive stages of S-ALI, HIF-1 activation in alveolar type II epithelial cells becomes essential for epithelial cell proliferation and tissue regeneration.

### 3.4 HIF-1 and oxidative stress in S-ALI

**Figure 2:**
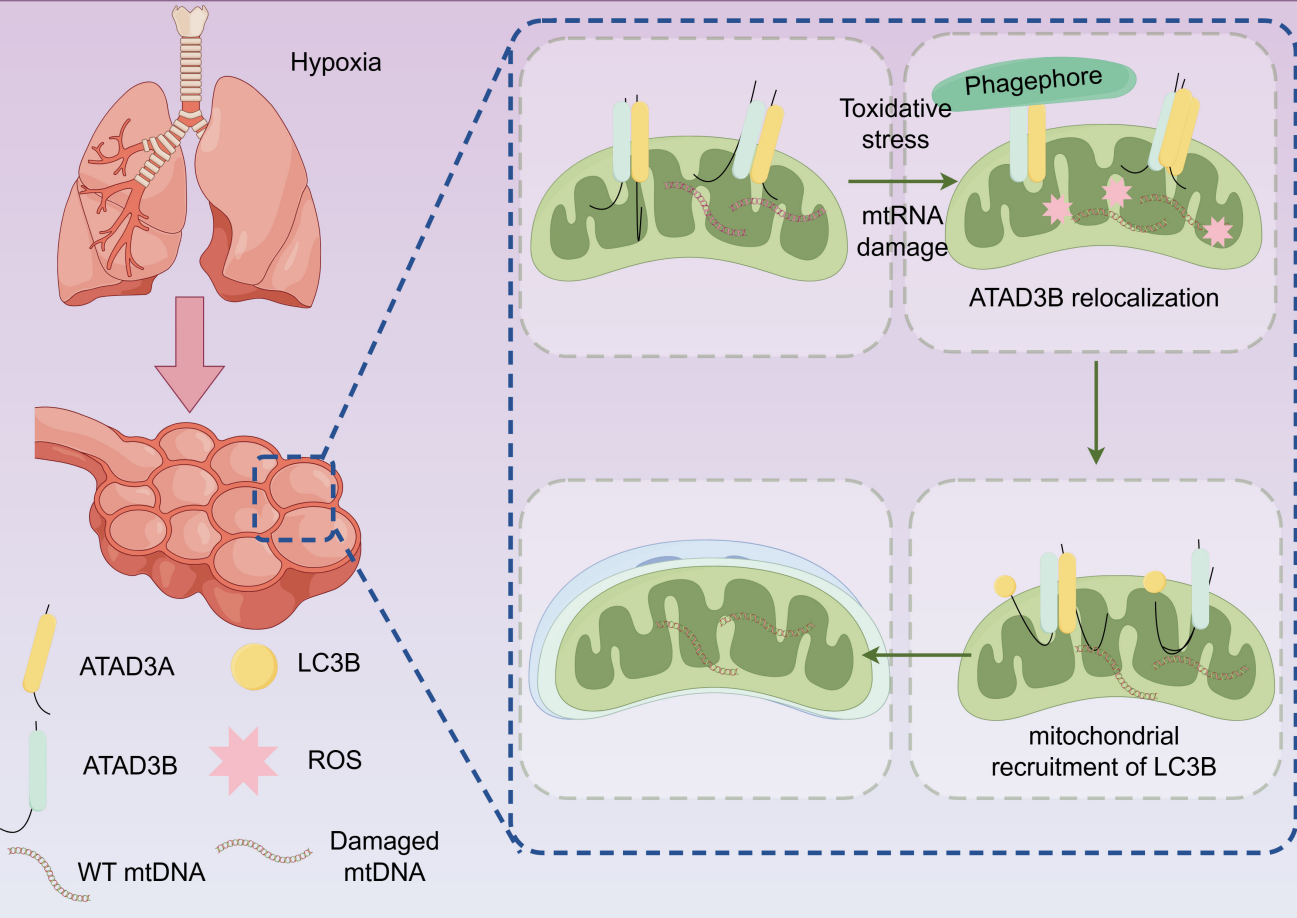
Oxidative stress and mitochondrial damage in S-ALI. Hypoxia leads to mitochondrial dysfunction in alveolar epithelial cells, oxidative stress, increased release of ROS, and further mtDNA damage and mitochondrial autophagy.ATAD3A, ATPase family AAA domain-containing protein 3A; ATAD3B, ATPase family AAA domain-containing protein 3B; LC3B, microtubule-associated protein 1 light chain 3B; ROS, reactive oxygen species; WT mtDNA, wild-type mitochondrial DNA.

During inflammatory responses, cellular energy metabolism intensifies, oxygen consumption increases, and reactive oxygen species (ROS) production escalates. This creates an imbalance between oxygen supply and demand, leading to relative tissue hypoxia and progressive mitochondrial dysfunction. The release of cytokines triggered by concurrent infection promotes ROS production, which causes lipid peroxidation and DNA damage to cell membranes (**Figure 2**), ultimately leading to cell necrosis or apoptosis. This oxidative stress state exacerbates alveolar epithelial cell damage, contributing to S-ALI development. ROS can also compromise the alveolar-vascular endothelial barrier structure, resulting in barrier dysfunction.

The hypoxic environment promotes both HIF-1 synthesis and mitochondrial ROS generation. Notably, elevated ROS levels can stabilize HIF-1 protein under hypoxic conditions. Additionally, hypoxia enhances oxidative stress through mitochondrial ROS production, and increased ROS can upregulate HIF-1 expression. Upon HIF-1 activation, the HIF-1α/heme oxygenase-1 (HO-1) pathway is subsequently activated, mediating ferroptosis in epithelial cells (26, 27), which triggers further ROS accumulation (27–29). Furthermore, ferroptotic cells release various damage-associated molecular patterns (DAMPs), while ROS may also induce mitochondrial damage and intracellular redox imbalance, further aggravating oxidative stress injury (29, 30).

### 3.5 HIF-1-driven metabolic reprogramming in S-ALI

Metabolic reprogramming refers to the process whereby cells reprogram their metabolic pathways to adapt to environmental changes under specific physiological or pathological conditions (31). The regulatory mechanisms governing metabolic reprogramming involve numerous signaling pathways and regulatory factors, with HIF-1α identified as a key mediator of monocyte metabolic reprogramming during sepsis (32). In septic states, the body experiences relative hypoxia, frequently accompanied by mitochondrial dysfunction characterized primarily by impaired oxidative phosphorylation and enhanced anaerobic glycolysis. This metabolic reprogramming affects cellular energy production and function, with S-ALI further exacerbating systemic hypoxia.

**Figure 3:**
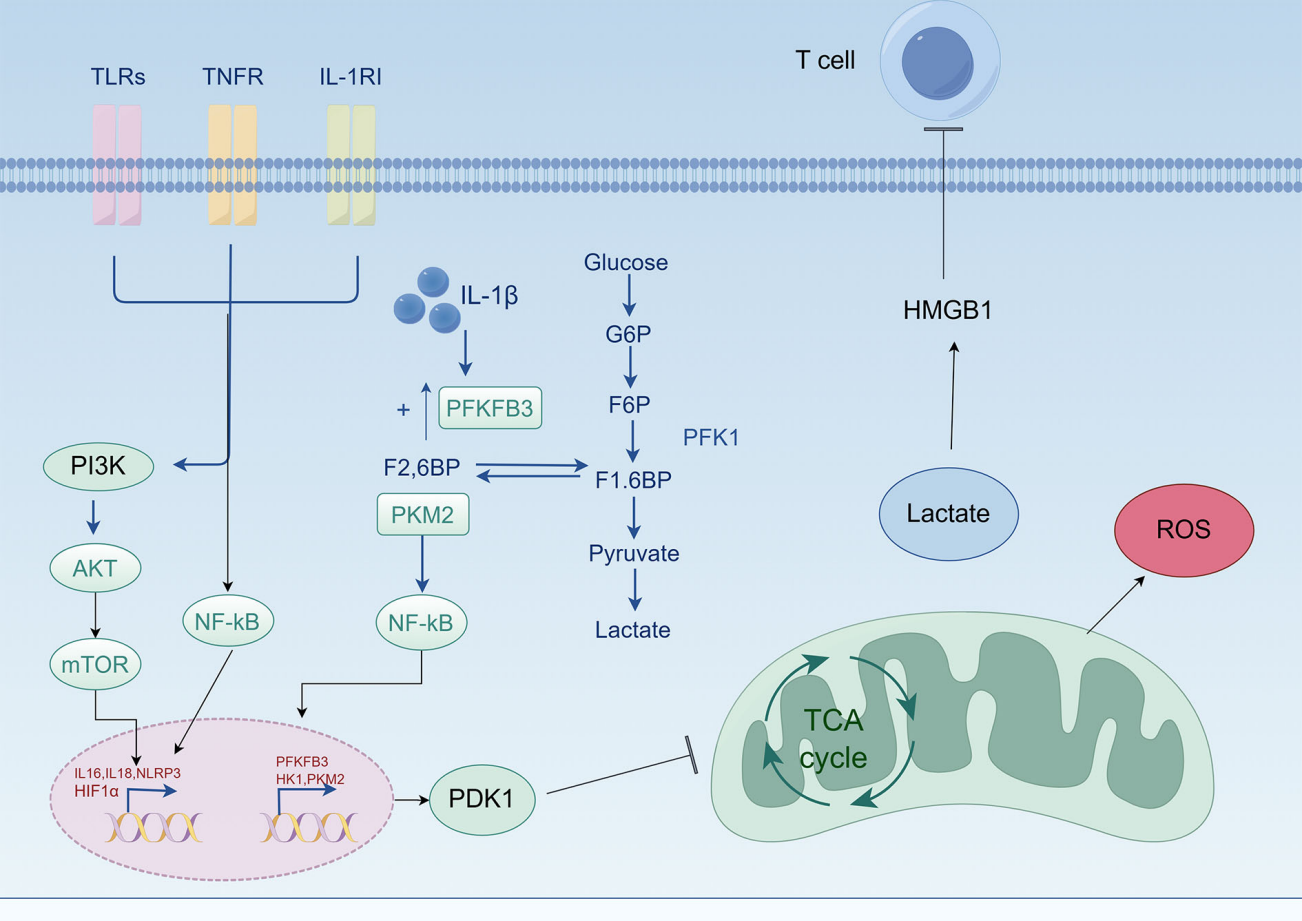
HIF-1 promotes glycolysis and inhibits oxidative phosphorylation. During sepsis, pattern recognition receptor proteins on lung tissue cells, such as TLRs, TNFR, and IL-1R, are activated by the source of infection, which in turn activates pathways such as PI3K/AKT/mTOR and NF-κB, intensifying inflammation and further exacerbating cellular hypoxia. This stabilizes HIF-1α, which translocates to the nucleus to synthesize HIF-1 and activate downstream inflammatory pathways such as NLRP3, as well as the expression of HK and PKM2, inhibiting the tricarboxylic acid cycle. Lactate dehydrogenase is activated to generate lactate, adapting to the hypoxic environment. Lactate further activates HMGB1 and inhibits T cell function. Meanwhile, hypoxia leads to oxidative stress and the production of ROS, causing damage to cells. Lactate released into the extracellular environment acidifies it, leading to cell apoptosis.TLRs, Toll-like receptors; TNFR, tumor necrosis factor receptor; IL-1R, interleukin-1 receptor; PI3K, phosphatidylinositol 3-kinase; AKT, protein kinase B; mTOR, mechanistic target of rapamycin; NF-κB, nuclear factor-κB; IL-6, interleukin-6; IL-18, interleukin-18; NLRP3, NOD-like receptor protein 3; HIF-1α, hypoxia-inducible factor-1α; IL-1β, interleukin-1β; F2, 6BP, fructose-2, 6-bisphosphate; HK2, hexokinase 2; PKM2, pyruvate kinase isoenzyme M2; PFKFB3, 6-phosphofructo-2-kinase/fructose-2, 6-bisphosphatase 3; PDK1, pyruvate dehydrogenase kinase 1; G6P, glucose 6-phosphate; F6P, fructose 6-phosphate; F1, 6BP, fructose-1, 6-bisphosphate; TCA cycle, tricarboxylic acid cycle; ROS, reactive oxygen species; HMGB1, high mobility group box 1.

During the hyperinflammatory phase of sepsis, HIF-1 activates lactate dehydrogenase A (LDHA), increasing lactate production and glycolytic flux. This shifts metabolism from oxidative phosphorylation to glycolysis, enabling cells to generate ATP through glycolysis and maintain viability even under conditions of insufficient oxygen supply (33). This metabolic adaptation enhances cell survival during crisis states (34). Moreover, this metabolic reprogramming not only provides a rapid energy source for immune cells but also promotes their survival and function, facilitating adaptation to hypoxic environments (35–37) (**Figure 3**).

#### 3.5.1 HIF-1 promotes glycolysis

HIF-1 enhances the glycolytic pathway by regulating expression of glycolysis-related enzymes (38). For instance, HIF-1 upregulates expression of hexokinase 2 (HK2), a key glycolytic enzyme responsible for converting glucose to glucose-6-phosphate, thereby channeling glucose into the glycolytic pathway (39). As glycolysis increases, lactate production rises proportionally. HIF-1 also upregulates LDHA expression, the key enzyme catalyzing pyruvate-to-lactate conversion. This process enables rapid pyruvate conversion to lactate under hypoxic conditions, preventing pyruvate accumulation and facilitating rapid energy production (40, 41).

Lactate accumulation increases microenvironmental acidity, potentially affecting cellular function and immune responses. In sepsis, for example, lactate overproduction is associated with tissue hypoxia, cellular damage, and inflammatory responses. By enhancing glycolysis, HIF-1 provides essential ATP to cells, enabling them to survive hypoxia and other stress conditions (42).

#### 3.5.2 HIF-1 inhibits oxidative phosphorylation

HIF-1α activation reduces the mitochondrial oxygen consumption rate (OCR), suggesting that under hypoxic conditions, cells may decrease their oxygen dependence and instead rely on anaerobic metabolic pathways for ATP production (43). During acute hypoxia, HIF-1α activation leads to increased mitochondrial ROS production. ROS functions not only as a metabolic byproduct but also as a signaling molecule regulating cellular physiology and pathology (44). Under HIF-1α regulation, cells adapt to hypoxic environments and maintain survival by modifying energy metabolic pathways. For example, HIF-1α not only inhibits fatty acid oxidation but also enhances glycolysis to meet cellular oxygen demands in hypoxic environments (45, 46).

### 3.6 HIF-1 regulation of immune cell function in S-ALI

#### 3.6.1 HIF-1 and macrophages

HIF-1 plays a crucial role in regulating immune cell function, particularly in macrophages (37). Macrophage function is closely associated with phenotype. Two major macrophage subtypes have been identified: M1 macrophages, which are pro-inflammatory or classically activated macrophages induced by interferon-γ (IFN-γ) and TNF-α, expressing high levels of pro-inflammatory cytokines; and M2 macrophages, which are anti-inflammatory macrophages induced by IL-4 and IL-13, contributing to inflammation resolution and injury repair. The roles of M1 and M2 macrophages in S-ALI are closely linked to HIF-1α (47).

During pro-inflammatory responses, M1 macrophages exhibit markedly increased glucose catabolism, predominantly dependent on HIF-1α and 6-phosphofructo-2-kinase expression, with pro-inflammatory activity proportionally regulated (48). In early S-ALI, stable HIF-1 expression in hypoxic environments promotes macrophage recruitment, activates glucose transporter 1 (GLUT-1) and hexokinase expression, enhances fluorodeoxyglucose uptake, upregulates glycolysis, and promotes inflammatory progression (49, 50). Conversely, restoring PHD2/HIF-1α signaling can inhibit M1 macrophage activation in S-ALI animal models, thereby suppressing pulmonary inflammation and improving outcomes (37). Studies have shown that ω-alkynylarachidonic acid inhibits HIF-1α binding to HRE sequences in the iNOS promoter and prevents pyruvate kinase isoenzyme M2 (PKM2) overexpression and nuclear translocation in macrophages during S-ALI. Regulating the PKM2-HIF-1α-iNOS interaction can promote M2 macrophage polarization and suppress inflammation (11, 51, 52).

#### 3.6.2 HIF-1 and neutrophils

During infection or inflammation, neutrophils are recruited to lesion sites where they release neutrophil extracellular traps for immune defense (14). Neutrophil mitochondrial metabolism and tricarboxylic acid cycle metabolism contribute to the survival and antibacterial functions of neutrophils and macrophages in hypoxic tissues. The prolonged survival of neutrophils under hypoxia is also dependent on HIF-1α expression (53).

In hypoxic infected tissues, HIF-1 enables these immune cells to adapt to environmental changes, maintain metabolic and functional activities, and thereby enhance immune function (54, 55). Concurrently, HIF-1 mediates phenotypic and functional reprogramming of monocytes during sepsis, transitioning them from an inflammatory to an immunosuppressive state, thereby attenuating inflammatory responses (56). HIF-1 also plays a vital role in regulating inflammatory responses. By promoting expression of specific inflammatory factors, HIF-1 can enhance immune cell function, facilitate pathogen clearance, and consequently reduce pulmonary inflammatory responses (20, 57). LDHA downregulation is a critical factor in inhibiting neutrophil glycolysis during S-ALI. The PI3K/Akt-HIF-1α pathway regulates LDHA expression levels, promotes glycolysis, and modulates neutrophil chemotaxis and phagocytosis (53).

### 3.7 HIF-1-mediated vascular endothelial remodeling in S-ALI

HIF-1 regulates vascular permeability by modulating endothelial cytoskeletal organization and intercellular junction mechanisms (58, 59). Alveolar epithelial cells and vascular endothelial cells constitute the alveolar-capillary barrier (60), and alveolar-capillary barrier permeability plays a pivotal role in S-ALI progression. Angiogenesis is essential for ALI recovery. VEGF is a critical angiogenic factor that stimulates endothelial cell proliferation and migration. As a transcription factor, HIF-1 regulates expression of downstream target genes including VEGF, EPO, and iNOS, inducing expression of proteins involved in glycolysis, angiogenesis, and cell survival, thereby promoting cellular adaptation to hypoxic environments (18, 61).

Under hypoxic conditions, the PI3K-Akt-mTOR signaling pathway may be activated in pulmonary artery endothelial cells, promoting pulmonary vascular remodeling (62, 63). A previous study has demonstrated that PI3K/AKT phosphorylation induces increased HIF-1α transcription under hypoxic conditions, with HIF-1 serving as a central regulator of post-hypoxic angiogenesis (64). Tissue hypoxia is a potent inducer of VEGF expression, accompanied by robust VEGF upregulation mediated by the transcription factor HIF-1.

During S-ALI, substantial plasma protein release and inflammatory cell activation occur, followed by relative pulmonary ischemia and hypoxia, triggering abundant HIF-1 synthesis. Through complex molecular mechanisms, HIF-1 interacts with the VEGF gene promoter region or related regulatory elements to promote VEGF expression and synthesis (10). VEGF binds to vascular endothelial growth factor receptor 2 (VEGFR2) on endothelial cell surfaces, thereby activating the autocrine VEGF/VEGFR2 signaling loop in endothelial cells (65). Consequently, the HIF-1/VEGF signaling pathway provides the necessary cellular foundation and molecular signaling support for new blood vessel formation, promoting tissue angiogenesis (66).

Angiogenesis facilitates restoration of the alveolar epithelial-endothelial barrier, reduces cytokine and inflammatory cell extravasation, and may alleviate lung tissue hypoxia (67). This phenomenon improves pulmonary microcirculation, reduces tissue hypoxia, promotes endothelial cell repair while reducing injury, thereby decreasing vascular permeability to some extent. These effects restore normal alveolar-capillary membrane function and promote acute lung injury repair.

### 3.8 Effects of HIF-1 on alveolar epithelium in S-ALI

Diffuse lung epithelial cell injury represents one of the principal mechanisms driving S-ALI pathogenesis. HIF-1 facilitates cellular adaptation to hypoxic environments and serves as a critical regulator of glucose metabolism in alveolar epithelial cells (AECs) during S-ALI (68). Studies have demonstrated that HIF-1α loss in the alveolar epithelium exacerbates lung injury during acute respiratory distress syndrome (ARDS), indicating that HIF-1α and the glycolytic pathway play pivotal roles in ALI (69). Alveolar epithelial barrier function is essential for preventing fluid and protein leakage from alveoli and maintaining optimal alveolar gas exchange. In alveolar epithelial cells, HIF-1 preserves alveolar epithelial barrier integrity by regulating numerous barrier function-related genes. Among these cells, alveolar type II epithelial cells play a crucial role in acute lung injury repair (24).

Alveolar type II epithelial cells enhance glycolysis through the HIF-1α-phosphofructokinase-2/fructose-2,6-bisphosphatase 3 (PFKFB3) axis, which subsequently amplifies glycolysis and promotes lactate release (70). In the lactate-rich microenvironment produced by alveolar type II epithelial cells, pulmonary macrophages undergo phenotypic switching from pro-inflammatory to anti-inflammatory states and exhibit increased expression of anti-inflammatory markers including IL-10 and arginase-1 (Arg-1) (71). Consequently, epithelial PFKFB3 enhances alveolar integrity by augmenting glycolytic metabolism during ALI, which is essential for ALI recovery (70). Stromal cell-derived factor-1 (SDF-1) and its receptor CXCR4 are target genes expressed in alveolar type II epithelial cells. HIF-1 upregulation during lung injury enhances AT-II cell migration *in vitro* and promotes barrier function restoration *in vivo (*72). Thus, HIF-1α has been shown to play a significant role in promoting alveolar epithelial proliferation.

The proliferative and reparative capacity of alveolar epithelial cells is crucial for lung tissue recovery following ALI. HIF-1α participates in regulating ATP synthesis, cell proliferation, and apoptosis in AECs. HIF-1α can stimulate AT-II cell proliferation and differentiation by upregulating growth factor expression and enhancing glycolysis, thereby promoting alveolar epithelial repair (73). However, increased HIF-1 expression is often accompanied by pathological AEC damage. In S-ALI, HIF-1α enhances glycolytic activity and acidifies the extracellular environment, consequently promoting cell apoptosis (74). Additionally, prolonged elevation of HIF-1 levels may induce pulmonary fibrosis formation. Alveolar epithelial cells can increase mitochondrial ROS levels, and ROS can upregulate both HIF-1α and TGF-β1 expression, thereby promoting epithelial-mesenchymal transition (9, 75).

## 4 HIF-1 and prognosis of sepsis

Studies have demonstrated that alterations in HIF-1 expression may be closely associated with clinical manifestations and prognosis of sepsis (76). First, HIF-1 expression changes significantly during sepsis. In one study, HIF-1α levels were generally elevated in septic patients, with this increase strongly correlated with the immune response triggered by bacterial infection (8). For instance, HIF-1α plays a critical role in the immune response to Streptococcus infection, suggesting that HIF-1 activation may represent an adaptive response to infection. However, other studies have revealed that both HIF-1α mRNA and protein levels are decreased in leukocytes of septic patients, with this decrease inversely correlating with sepsis severity. This implies that reduced HIF-1α levels may be associated with more severe sepsis, suggesting that HIF-1α expression may reflect the body’s capacity to respond to pathological conditions.

In sepsis, HIF-1 is stimulated not only by hypoxia but also by inflammatory processes. Studies have shown that HIF-1 activation is closely linked to intracellular hypoxia and systemic inflammatory responses (8). In the septic context, HIF-1 facilitates cellular adaptation to hypoxia by regulating multiple metabolic pathways. This metabolic reprogramming can enhance cell viability; however, dysregulated HIF-1 activity can lead to tissue damage and organ dysfunction.

**Figure 4:**
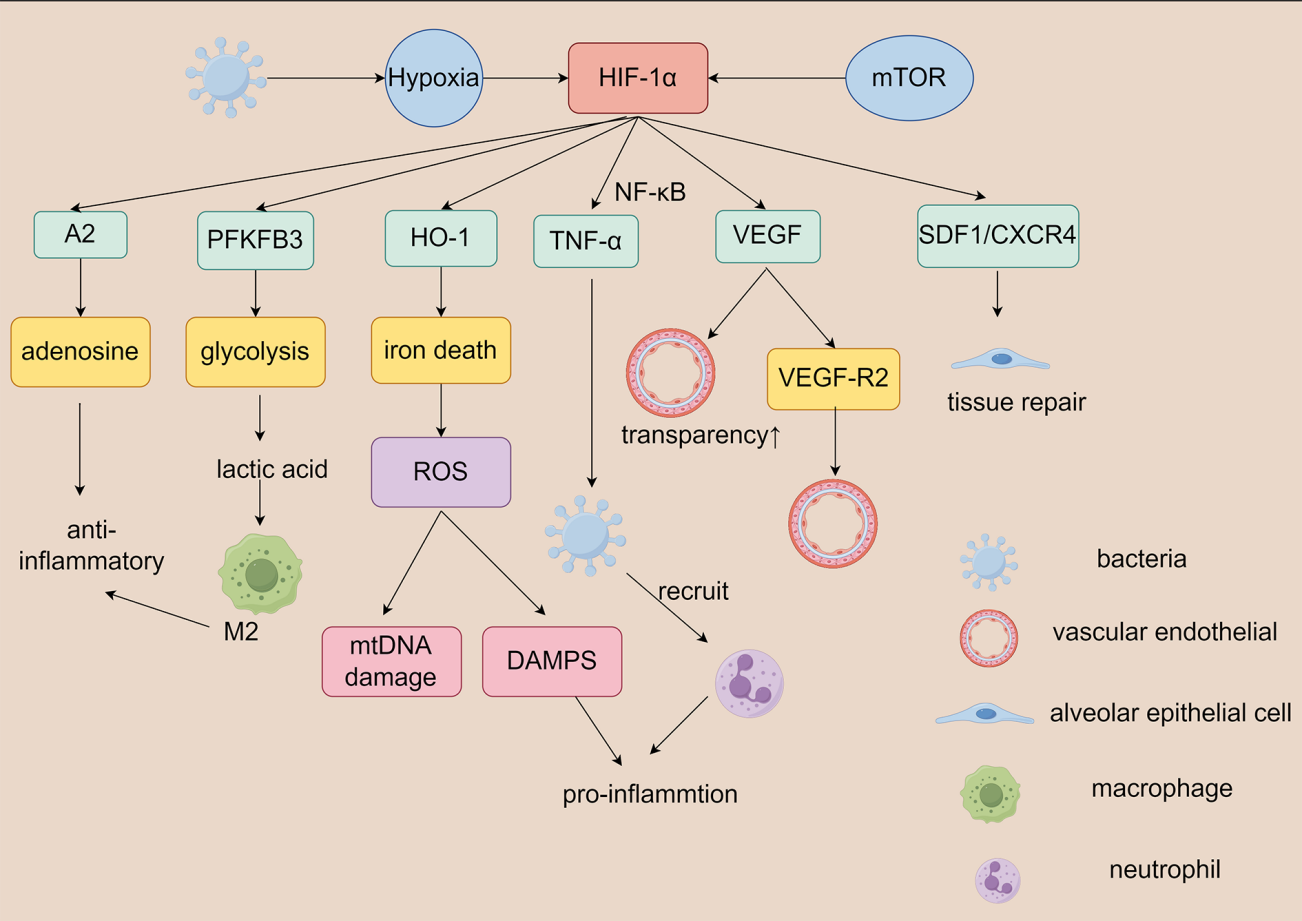
Role of HIF-1 in metabolic reprogramming of sepsis-associated lung injury.During the sepsis, pathogen-induced hypoxia stabilizes the expression of HIF-1α in pulmonary tissue cells and facilitates the activation of downstream signaling pathways, thereby modulating the functions of relevant cells—including macrophages, neutrophils, alveolar epithelial cells, and vascular endothelial cells. HIF-1α, hypoxia-inducible factor-1α; ROS, reactive oxygen species;PFKFB3, 6-phosphofructo-2-kinase/fructose-2, 6-bisphosphatase 3; A2, Adenylate kinase-2; HO-1, heme Oxygenase-1;TNF-α, tumor necrosis factor-α;NK-κB, nuclear factor-κB;VEGF, vascular endothelial growth factor;VEGF-R2, vascular endothelial growth factor-receptor2;mTOR, mammalian target of rapamycin;SDF1, Stromal Cell-derived Factor 1;CXCR4, CXC chemokine receptor4;mtDNA, mitochondrial DNA.

Furthermore, HIF-1 expression in leukocytes of septic patients has been shown to correlate directly with patient survival, suggesting that HIF-1 expression may serve as a prognostic indicator for sepsis (55). This association underscores HIF-1’s importance in sepsis and may provide novel biomarkers for clinical application. In the context of S-ALI, HIF-1’s role in metabolic reprogramming has been extensively investigated. HIF-1 not only regulates glycolysis but also influences fatty acid metabolism and amino acid metabolism, thereby promoting cell survival under hypoxic conditions (35). This metabolic adaptation may exert protective effects in sepsis-induced acute lung injury but may also contribute to pathological state exacerbation (**Figure 4**).

## 5 Potential applications of HIF-1 in treating sepsis-induced lung injury

Modulating HIF-1 activity can improve overall clinical outcomes in septic patients. HIF-1 regulation may reduce pulmonary inflammation, enhance oxygenation, strengthen host responses to infection, and consequently decrease the incidence and severity of acute lung injury (77). HIF-1’s potential as a therapeutic target is primarily reflected in its capacity to regulate various biological pathways, including NF-κB, JAK2/STAT3, mTOR, Notch, and MAPK, which are critical in the pathogenesis of sepsis and acute lung injury (5, 65). Additionally, studies investigating inhibitors of catalytic enzymes required for HIF-1 activation may prove valuable. Previous research has demonstrated that the HIF-1 prolyl hydroxylase inhibitor roxadustat (FG-4592) attenuates sepsis-induced acute lung injury (78). Drugs targeting HIF-1 may provide synergistic therapeutic effects by modulating multiple pathways, including emodin (65, 79), COMP-Ang1 (79, 80), Roxadustat (80), DMOG(HIF-1α stabilizer) (81), dexmedetomidine (30), melatonin (50), and Shenfu injection (82)(**Table 1**). With enhanced understanding of HIF-1 mechanisms, individualized treatment approaches will become increasingly feasible. Different patients may exhibit varied responses to HIF-1 modulation, and research could identify patient populations that respond favorably to specific HIF-1 inhibition or activation therapies, thereby optimizing treatment strategies.

**Table 1:** Therapeutic interventions and mechanisms of S-ALI targeting HIF-1.

Treatment agent	Function and targets
Emodin	Downregulation of multiple inflammatory cytokines via the mTOR/HIF-1α/VEGF signaling pathway,induced S-ALI
COMP-Ang1	Inhibition of HIF-1α significantly mitigates lung injury in a H_2_O_2_-induced murine model of S-ALI
Roxadustat	Therapeutic intervention of HIF-1α-dependent suppression attenuates pulmonary epithelial cell injury.
DMOG	Enhanced HIF-1α-dependent glycolysis confers protection against LPS-induced lung injury.
dexmedetomidine	Preserving mitochondrial dynamic equilibrium through the HIF-1a/HO-1 signaling pathway against LPS-induced lung injury.
melatonin	Regulates the activation of the ROS/HIF-1α/GLUT1/NLRP3 pathway in alveolar macrophages alleviating S-ALI
Shenfu	Promote mitophagy to improve mitochondrial function by regulating the expression of HIF-1α

COMP-Ang1, angiopoietin-1 chimeric protein; S-ALI, sepsis-associated lung injury; LPS, Lipopolysaccharide; H_2_O_2_, hydrogen peroxide; mTOR, mammalian target of rapamycin; HIF-1α, hypoxia-Inducible Factor-1α; VEGF, vascular endothelial growth factor; DMOG, prolyl hydroxylase inhibitor; HO-1, heme Oxygenase-1; ROS, reactive oxygen species; GLUT1, glucose transporter 1; NLRP3, NOD-like receptor protein 3.

However, it is important to note that HIF-1 not only promotes inflammatory progression in S-ALI but also plays divergent roles at different disease stages. On one hand, it promotes inflammation; on the other hand, it facilitates adaptation to and tolerance of hypoxia while promoting endothelial barrier repair and proliferation. These dual effects may exert both positive and negative influences on physiological processes. Therefore, the safety of HIF-1-targeted therapies must be carefully evaluated during drug development to avoid adverse effects on other physiological processes.

HIF-1 activity is influenced by multiple factors, including oxygen concentration, cell type, and microenvironmental conditions. This complexity in HIF-1’s role across different tissues and pathological states poses challenges for targeted therapies. Whether HIF-1α ultimately exacerbates injury or provides protection (or both) requires additional basic research to clarify HIF-1’s specific roles under different circumstances. Clinical trial design for HIF-1-targeted therapies must consider numerous variables, including patients’ underlying diseases, routes of administration, and treatment timing. The complexity of these factors may influence clinical trial outcomes, potentially delaying HIF-1’s application as a therapeutic target.

HIF-1’s regulatory role in fatty acid metabolism and oxidative phosphorylation is critical for understanding metabolic reprogramming in pathological states such as sepsis and acute lung injury. HIF-1α facilitates adaptation to hypoxic and inflammatory environments by inhibiting fatty acid β-oxidation, reducing lipid mobilization and uptake, affecting mitochondrial function, and altering energy metabolic pathways. These mechanisms not only support cell survival and function but may also represent novel therapeutic targets for related diseases.

Overall, HIF-1 holds substantial promise as a potential therapeutic target for sepsis and acute lung injury, with capacity to improve clinical outcomes through multiple mechanisms, including metabolic reprogramming, inflammatory response regulation, and vascular repair. However, developing safe and effective HIF-1-targeted therapies faces numerous challenges, including potential side effects, biological complexity, and clinical trial design considerations. Therefore, advancing HIF-1-targeted therapy requires strengthening the integration of basic research and clinical trials to provide more effective treatment options for patients with sepsis and acute lung injury.

## 6 Conclusions

HIF-1 plays multifaceted roles in sepsis-induced acute lung injury through metabolic reprogramming, including regulation of inflammatory responses, immune cell function, and cellular metabolic pathways. In-depth understanding of HIF-1 mechanisms not only helps elucidate the pathological processes underlying acute lung injury but also provides crucial foundations for developing novel therapeutic strategies. Future studies should further investigate HIF-1’s specific regulatory mechanisms in lung injury and explore its potential clinical applications.
